# Chlorogenic Acid Inhibits Ceramide Accumulation to Restrain Hepatic Glucagon Response

**DOI:** 10.3390/nu15143173

**Published:** 2023-07-17

**Authors:** Na Xiao, Tengfei Zhang, Mingli Han, Dan Tian, Jiawei Liu, Shan Li, Lele Yang, Guojun Pan

**Affiliations:** 1 College of Agronomy, Shandong Agriculture University, Tai’an 271018, China; 2 State Key Laboratory of Quality Research in Chinese Medicine, Institute of Chinese Medical Sciences, University of Macau, Macau 999078, China; 3 College of Life Sciences, Shandong First Medical University & Shandong Academy of Medical Sciences, Tai’an 271000, China

**Keywords:** chlorogenic acid, glucagon, gluconeogenesis, ceramide, Akt/FoxO1

## Abstract

Chlorogenic acid (CGA), a dietary natural phenolic acid, has been widely reported to regulate glucose and lipid metabolism. However, the protective effects and the underlying mechanisms of CGA on glucagon-induced hepatic glucose production remain largely uncharacterized. Herein, we investigated the efficacy of CGA on hepatic gluconeogenesis both in vivo and in vitro. The elevated levels of endogenous glucose production induced by infusion of glucagon or pyruvate were lowered in mice administered with CGA. Furthermore, chronic CGA treatment ameliorated the accumulation of glucose and ceramide in high-fat diet (HFD)-fed mice. CGA also attenuated HFD-fed-induced inflammation response. The protective effect of CGA on glucose production was further confirmed in primary mouse hepatocytes by inhibiting accumulation of ceramide and expression of p38 MAPK. Moreover, CGA administration in HFD-fed mice preserved the decreased phosphorylation of Akt in the liver, resulting in the inhibition of FoxO1 activation and, ultimately, hepatic gluconeogenesis. However, these protective effects were significantly attenuated by the addition of C2 ceramide. These results suggest that CGA inhibits ceramide accumulation to restrain hepatic glucagon response.

## 1. Introduction

Type 2 diabetes mellitus (T2DM) is characterized by elevated blood glucose, which consists of up to 90% of all diabetes cases [1]. The dysfunction of insulin action leads to insulin resistance, resulting in abnormal plasma glucose concentration with increased glucose production and decreased glucose uptake [2]. Additionally, studies have well characterized the heterogeneous nature of T2DM, which accelerates the research and development of different classes of drugs to treat hyperglycemia [3]. It can be concluded that most of the widely applied treatments are drugs that directly or indirectly target insulin action. Among the approved drugs, metformin is widely applied, which mainly reduces hepatic gluconeogenesis to prevent hyperglycemia during fasting [4]. Evidence is accumulating that endogenous hepatic glucose production that is associated with the action of glucagon plays a critical role in the regulation of glucose homeostasis [5,6,7,8].

Glucagon increases hepatic glucose output through the activation of the cAMP/PKA pathway, upregulating the gene transcription of gluconeogenesis key rate-limiting enzymes such as glucose-6-phosphatase (G6pase) and phosphoenol pyruvate carboxykinase (PEPCK) [5]. Glucagon can also directly inhibit Akt phosphorylation (Ser473), block Akt’s inhibition of FoxO1 transcriptional activity, and promote FoxO1 into the nucleus, thereby upregulating the expression of G6pase and PEPCK and promoting hepatic sugar production [8]. In diabetes, dysregulated glucagon response induces excessive hepatic glucose output, contributing to fasting hyperglycemia [7,8,9,10]. Therefore, these studies suggested that the pharmacological regulation of Akt might block hepatic gluconeogenesis via FoxO1 phosphorylation and nuclear exclusion.

Ceramides are a class of lipids that play a central role in cell signaling, and studies have shown that ceramides are strongly associated with the development of metabolic diseases such as obesity and diabetes [10,11,12]. Knocking down dihydroceramide desulfurase 1 (Degs1) may improve insulin resistance and hepatic lipid accumulation in mice by reducing ceramide accumulation in mouse tissues, suggesting that ceramide accumulation is an important factor in causing disorders of glycolipid metabolism [11,12]. There are many studies between ceramides and disorders of glycolipid metabolism, but most of them focus on insulin resistance and lipid accumulation, while the relationship with glucagon-mediated responses has not been established. In the liver and adipose tissue, inflammation due to metabolic disorders promotes ceramide synthesis [13,14], while inflammatory signals participate in and amplify the glucagon response and promote hepatic glucose output [5,7,15]. When the regulation of metabolic stresses caused by overnutrition does not function properly, many pro-inflammatory and inflammatory cytokines such as TNF, IL-6, and IL-1β are produced, which leads to the elevated glucose production and subsequent high blood glucose [13,15]. These findings raise the question of whether inflammatory signals promote ceramide accumulation, and then ceramide accumulation leads to increased glucagon-mediated gluconeogenesis and hepatic glucose output. The development of drugs and supplementary food products consumed daily, especially supplementary food products consumed daily, that inhibit hepatic glucose production via regulation of inflammation is urgently needed.

Chlorogenic acid (CGA) is one of the most abundant phenolic acids in foods and edible and medicinal plants, such as coffee, *Lonicera japonica*, and *Crataegi fructus*, and its roles in the suppression of inflammation and oxidative stress are well-documented [16,17,18]. Emerging evidence demonstrates its ability to ameliorate metabolic disorders. CGA has also been shown to increase the expression of hepatic AMPK in protecting Lepr*^db/db^* mice and rats from abnormal blood glucose levels [19,20]. However, whether and how CGA regulates the metabolism of ceramide to restrain the hepatic glucagon response in diabetic mice remains largely uncharacterized. In this work, we investigated the effect of CGA on hepatic gluconeogenesis with focus on the regulation of metabolic alternations. Herein, both acute and high-fat diet (HFD)-induced mice models were used to investigate the effects of CGA on endogenous glucose production, inflammatory response, and lipid metabolism. Subsequently, we attempted to test whether the inhibitory action of CGA on hepatic glucagon response is linked to the suppression of ceramide accumulation and inflammatory response in primary mouse hepatocytes.

## 2. Materials and Methods

### 2.1. Chemicals and Reagents

Chlorogenic acid (purity ≥ 98%) was purchased from Solarbio Science & Technology Co., Ltd. (Beijing, China). Metformin and C2 ceramide were purchased from Sigma-Aldrich (St. Louis, MO, USA), and reconstituted glucagon was purchased from Novo Nordisk (Bagsvaerd, Denmark). The SB203580 was obtained from MedChemExpress company (Junction, NJ, USA).

### 2.2. Animals and Treatment

For this study, 6–8-week-old male C57BL/6J mice were offered from Pengyue Laboratory Animal Company (Jinan, China) and were housed in colony cages with a 12 h light–dark cycle. All experiments and animal care were conducted in accordance with the Provision and General Recommendation of Chinese Experimental Animals Administration Legislation approved by the Animal Ethics Committee of Shandong Agriculture University (SDAUA-2020-034, approval date: 30 March 2020). The mice were divided into chow group, HFD group, CGA group (150 mg/kg), and metformin group (200 mg/kg). Except for the chow group, all the mice were fed with HFD (D12492, Research Diets, Inc., New Brunswick, NJ, USA) for 12 weeks. The mice in the chow group were fed with regular chow diet. CGA and metformin group were given CGA and metformin orally once a day, respectively. Chow-fed group and HFD-fed group were given an equivalent volume of saline orally once a day for 12 weeks. The body weight and food intake of the mice were recorded weekly. Blood was collected after an overnight fasting, and metabolic variables were assayed with commercial kits (Jiancheng, Nanjing, China)

### 2.3. Glucagon, Pyruvate, and Oral Glucose Tolerance Tests

For glucagon tolerance test (GTT) and pyruvate tolerance test (PTT), C57BL/6J mice were fasted for 16 h and randomly divided into control group administered with saline, model group administered with saline, CGA group administered with 150 mg/kg of CGA, and metformin group administered with 200 mg/kg of metformin. One hour after administration, the mice were injected intraperitoneally with 2 mg/kg glucagon or 2 g/kg pyruvate to observe the changes of blood glucose within 2 h. For PTT, C2 ceramide was administered through intraperitoneal injection (120 μM) after CGA intervention to evaluate the effect of ceramide on gluconeogenesis. For oral glucose tolerance test (OGTT), the HFD-fed mice were administered 2 g/kg glucose solution. Blood was collected from tail tips at indicated times to assay glucose content. The area under the curve (AUC) was calculated for blood glucose using the following formula: 0.5 × (0 h blood glucose + 0.5 h blood glucose)/2 + 0.5 × (0.5 h blood glucose + 1 h blood glucose)/2 + (1 h blood glucose + 2 h blood glucose)/2.

### 2.4. Preparation of Primary Hepatocytes and Treatment

Primary hepatocytes were isolated from male C57BL/6J mice using an improved two-step collagenase infusion [5,7]. In brief, C57BL/6J mice starved for 16 h were anesthetized with 1% pentobarbital sodium. Then, the liver was digested with liquid I and liquid II containing type IV collagenase. Then, the primary hepatocytes were filtered, centrifuged, and cultured in DMEM supplemented with 10% FBS. Hepatocytes were maintained on 48-well plates or 6-well plates. After attachment, the media were replaced with serum-free medium to fast the cells. Then, the cells were treated with the indicated drugs in the presence or absence of 100 nM glucagon.

### 2.5. Glucose Output

Primary mouse hepatocytes were seeded in a 48-well plate. After attachment, the media were replaced with Krebs-Ringer HEPES buffer to fast the cells. Then, the cells were incubated with relevant substrates (10 mM pyruvate, 100 nM glucagon) or with metformin (1 mM), CGA (10, 100 μM), and SB203580 (10 μM) for 6 h. The cell supernatant was collected for glucose analysis using the Glucose Assay Kit.

### 2.6. Western Blot Analysis

Liver tissue or cell protein was extracted with RIPA buffer with protease or phosphatase inhibitors. After centrifugation, the supernatants were quantified, and 60–80 µg protein samples were separated using 8–10% SDS-PAGE gels and transferred onto PVDF membranes, blocked, and then incubated with primary antibodies and secondary antibody (Supplementary Table S1). The antibody reactivity was then detected using ECL and quantified with IPP 6.0 software.

### 2.7. Measurement of Ceramide, IL-6 and TNF-α

Primary mouse hepatocytes were cultured in 6-well plates and treated in indicated condition. After treatment, hepatocytes were washed, collected, and centrifuged to remove cellular debris. Liver tissues were rinsed, weighed, and lysed in cell lysis buffer. Then, the resulting suspension was subjected to ultrasonication and centrifugation. The supernatant was measured for ceramide (Jiangsu Meibiao Biotech, Nanjing, China), IL-6, and TNF-α (Chengbin Biotech, Shanghai, China) using ELISA following the manufacturer’s instructions.

### 2.8. Quantitative Real-Time PCR

Total RNA was isolated from cultured hepatocytes or liver tissue using Trizol reagent, and cDNAs were synthesized. Quantitative PCR was performed on the Roche LightCycler 96 System using the Fast SYBR Green Master Mix (Roche, Germany). The mRNA level of target genes, including *G6pase*, *PEPCK*, *Degs1, CerS5*, and *CerS6*, were normalized to *β-actin* and calculated using the ΔΔCT method. The sequences of primers used in this study are listed in Table 1.

### 2.9. Immunofluorescence

Primary hepatocytes were incubated with CGA (100 μM), C2ceramide (100 μM), or SB203580 (10 μM) for 4 h, then stimulated with glucagon (100 nM) for 1 h. After treatment, hepatocytes were washed with ice-cold PBS and fixed with 4% paraformaldehyde. Then, the cells were permeabilized and blocked with 3% BSA including 0.3% Triton X-100, followed by incubation with anti-FoxO1 primary antibodies overnight at 4 °C. After several washings, the cells were incubated with second antibody in the dark for 1 h and were incubated with DAPI for 15 min. The image was visualized under a confocal scanning microscope.

### 2.10. Statistical Analysis

All results are expressed as the means ± SD. Comparisons between the two groups were analyzed with Student’s *t*-test, and more than two groups were analyzed with one-way ANOVA followed by Tukey’s test (GraphPad Prism 6.0 software). A value of *p* < 0.05 was considered statistically significant.

## 3. Results

### 3.1. CGA Inhibits Endogenous Glucose Production in Mice

**Figure 1:**
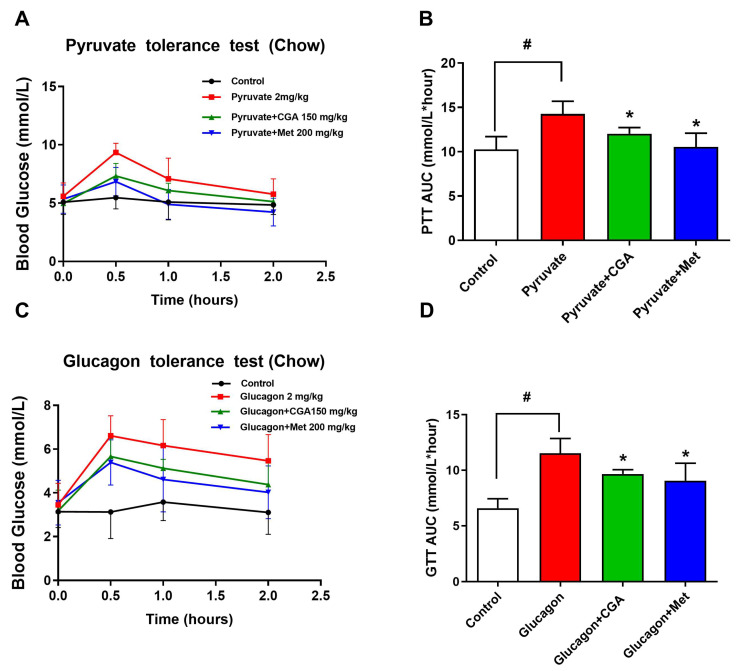
CGA-inhibited endogenous glucose production in mice. After 1 h of gavage administration of indicated drugs, glucagon (2 mg/kg) or pyruvate (2 g/kg) was injected intraperitoneally, and blood glucose levels and AUC were measured in pyruvate tolerance test (PTT) (**A**,**B**) and glucagon tolerance test (GTT) (**C**,**D**). Data were expressed as the mean ± SD (n = 8). * *p* < 0.05 vs. Pyruvate, Glucagon, ^#^ *p* < 0.05 vs. Control. CGA, Chlorogenic acid; Met, Metformin.

Fasting blood glucose is mainly regulated by endogenous glucose production, while pyruvate tolerance test is an indicator of glucose production. Thus, we examined whether CGA could improve pyruvate tolerance in C57BL/6J mice. The results showed that the hyperglycemic response to pyruvate was significantly higher in the pyruvate group than in the control group, whereas this alternation was reversed by CGA treatment (Figure 1A,B). CGA treatment attenuated the hyperglycemic response to glucagon challenge in normal mice (Figure 1C,D), and metformin as a positive control showed a similar regulation as CGA. These results suggested that CGA inhibited endogenous glucose production via restraining hepatic glucagon response.

### 3.2. CGA Inhibits Hepatic Glucagon Response and Inflammation Response

**Figure 2:**
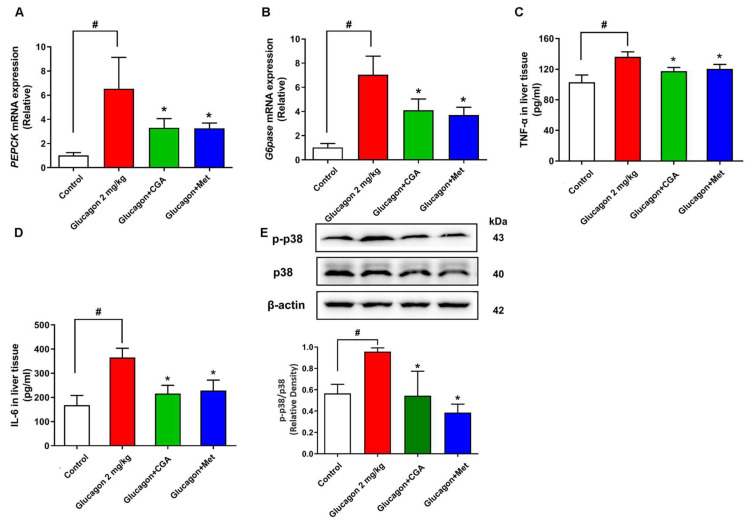
CGA-attenuated hepatic glucagon response and inflammation response in mice. CGA inhibited mRNA levels of *PEPCK* (**A**) and *G6pase* (**B**) in the liver (n = 4). CGA inhibited glucagon-induced TNF-α, IL-6 production (n = 6) (**C**,**D**) and p-p38 protein expression (**E**) (n = 4). Data are expressed as the mean ± SD. * *p* < 0.05 vs. Glucagon, ^#^ *p* < 0.05 vs. Control. CGA (150 mg/kg), Chlorogenic acid; Met (200 mg/kg), Metformin.

Hepatic gluconeogenesis-related genes were examined to validate the actions of CGA on hepatic glucagon response. As shown in Figure 2A,B, glucagon increased the gene expression of *PEPCK* and *G6pase* by about 4-fold in mice liver. However, these effects of glucagon were attenuated by the administration of 150 mg/kg CGA. As expected, the mRNA expression of *PEPCK* and *G6pase* was strongly inhibited by 200 mg/kg metformin (Figure 2A,B), confirming the blood glucose lowering effect via hepatic gluconeogenesis. It is important to note that the upregulation of inflammation play an important role in hepatic glucagon response, and CGA has been found to alleviate inflammatory response. The total levels of main inflammation indicators were quantified in liver tissues. The results showed that glucagon increased the levels of TNF-α, IL-6, and p-p38 MAPK in the liver; however, these effects were reversed by the administration of CGA or metformin accompanied by reduced glucose levels (Figure 2C–E).

### 3.3. CGA Inhibits Expression of Ceramide Synthase 6 and Ceramide Accumulation

**Figure 3:**
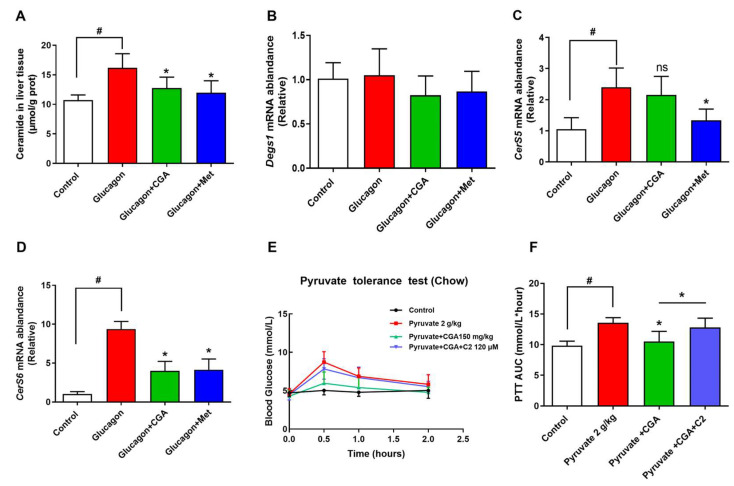
CGA-attenuated *CerS6* expression and ceramide accumulation in mice. After glucagon (2 mg/kg) stimulation, the effects of CGA on ceramide accumulation (n = 6), mRNA levels of dihydroceramide dehydrogenase 1 (*Degs1*) and ceramide synthase (*CerS5*, *CerS6*) in mice liver tissues were observed (**A**–**D**) (n = 4). C2-Ceramide (C2, 120 μM) was injected intraperitoneally in C57BL/6J mice, and blood glucose levels and AUC were measured to observe the effect of CGA on pyruvate tolerance (**E**,**F**) (n = 8). Data are expressed as the mean ± SD. * *p* < 0.05 vs. Glucagon or pyruvate, ^#^ *p* < 0.05 vs. Control. ^ns^ *p* > 0.05. CGA (150 mg/kg), Chlorogenic acid; Met (200 mg/kg), Metformin.

We next explored the potential mechanism by which CGA restrains hepatic glucagon response. p38 MAPK has been shown to activate the ceramide synthesis, and ceramide has been implicated in liver tissues to play a key role in causing dysregulation of glucagon response. As shown in Figure 3A, glucagon significantly upregulated ceramide accumulation in mice liver. The mRNA levels of main proteins involved in ceramide synthesis were tested, including *Degs1*, *ceramide synthetase* (*CerS*) *5*, *CerS6* (Figure 3B–D). The results indicated that glucagon elevated *CerS6* expression. In contrast, *CerS6* expression and ceramide accumulation were decreased in the liver of CGA-administered mice. To test the hypothesis more directly, short-chain cell permeable C2 ceramide was employed in a pyruvate tolerance test. The inhibition effect of CGA on the hepatic glucose output was abolished in the mouse administered with C2 ceramide (Figure 3E,F), suggesting the important role of inflammation-induced ceramide accumulation in hepatic glucagon response.

### 3.4. CGA Inhibits Glucose Output and Ceramide Accumulation in Primary Hepatocytes

**Figure 4:**
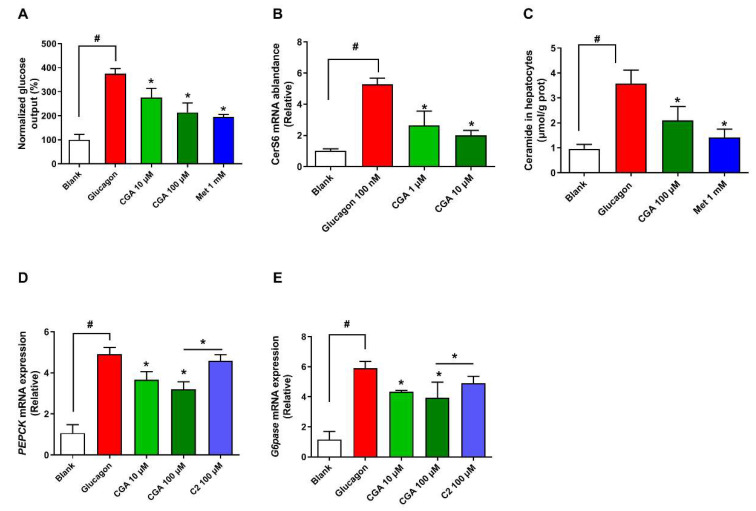
CGA inhibits glucose output and ceramide accumulation in primary hepatocytes. After stimulation with glucagon (100 nM), the effects of CGA on glucose output, the mRNA levels of *CerS6* (n = 4), and ceramide accumulation (**A**–**C**) were observed in primary hepatocytes. The effect of C2-ceramide (C2) on mRNA levels of *PEPCK* (**D**) and *G6pase* (**E**) in primary hepatocytes (n = 4). Data are expressed as the mean ± SD (n = 6). * *p* < 0.05 vs. Glucagon, ^#^ *p* < 0.05 vs. Glucagon. CGA, Chlorogenic acid; Met, Metformin.

Mouse primary hepatocytes were employed to further determine the role of inflammation-induced ceramide in hepatic gluconeogenesis. Consistent with the results of in vivo glucose output, the level of *CerS6* mRNA and ceramide was enhanced by glucagon, which was suppressed in the presence of CGA (Figure 4A–C). The change in mRNA expression of *PEPCK* and *G6pase* was similar to that of glucose production (Figure 4D,E). However, the repressed glucose production as well as expression of PEPCK and G6pase in hepatocytes treated with CGA was reversed by the addition of C2 ceramide (Figure 4D,E). The above results indicate that CGA restrain glucagon response in part via the regulation of ceramide accumulation.

### 3.5. CGA Protects Akt Phosphorylation and Inhibits FoxO1 Activation in Hepatocytes

**Figure 5:**
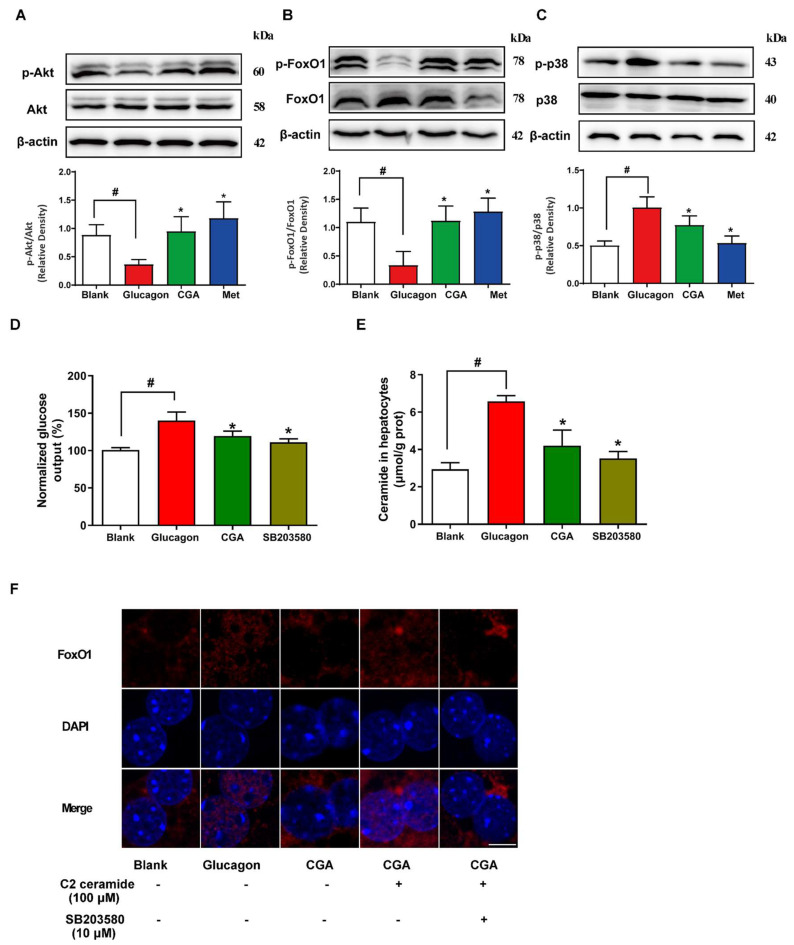
The effect of CGA on Akt/FoxO1 pathway in hepatocytes. After stimulation with glucagon (100 nM), the effects of CGA (100 μM) and Met (1 mM) on protein expression of Akt and FoxO1 and phosphorylation of p38 and FoxO1 (**A**–**C**) in hepatocytes were analyzed by western blot. Primary mouse hepatocytes were pretreated with CGA in the absence or presence of C2 ceramide or SB203580, followed by incubation with glucagon for 6 h. The effects of CGA on glucose output and ceramide accumulation (**D**,**E**) were observed in primary hepatocytes. The image of FoxO1 nucleus translocation was viewed with confocal scanning microscope (**F**) (Red: FoxO1; Blue: DAPI). Scale bar: 5 μm. Data are expressed as the mean ± SD (n = 4). * *p* < 0.05 vs. Glucagon, ^#^ *p* < 0.05 vs. Blank. CGA, Chlorogenic acid; Met, Metformin.

CGA has been reported to regulate glucose metabolism via the phosphorylation of Akt. Thus, the level of Akt phosphorylation was examined to further clarify the underlying mechanisms by which CGA inhibited gluconeogenesis through the regulation of ceramide accumulation. In hepatocytes, glucagon lowered the activity of Akt by reducing its phosphorylation levels, whereas administration of CGA restored the expression of Akt phosphorylation (Figure 5A). The activation of Akt inhibits hepatic glucagon response by inhibiting glucagon-mediated FoxO1 activation in hepatocytes. Consistently, glucagon increased the protein expression of FoxO1, phosphorylation p38, and reduced FoxO1 phosphorylation expression, but these alternations were reversed by CGA and metformin treatment (Figure 5B,C). In hepatocytes, glucagon induced glucose production and ceramide accumulation, whereas these alterations were prevented by incubation with CGA or p38 MAPK inhibitor SB203580, indicative of the potential role of p38 in the production of ceramide (Figure 5D,E). Consistently, the view of confocal microscope showed that CGA effectively blocked glucagon-induced nuclear translocation of FoxO1, but this change was reversed by pretreatment with C2 ceramide (Figure 5F). However, SB203580 reversed the effect of C2 ceramide on nuclear translocation of FoxO1, suggesting that the inhibitory effect of CGA on p-p38 expression contributed to reducing ceramide accumulation, leading to decreased glucose production.

### 3.6. CGA Inhibited Endogenous Glucose Production and Ceramide Accumulation in HFD-Fed Mice

**Figure 6:**
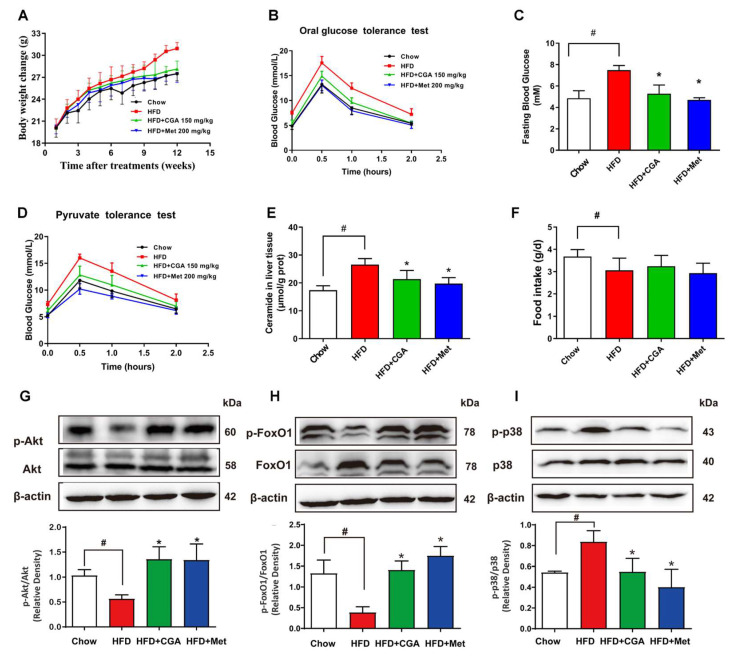
The effect of CGA on endogenous glucose production and ceramide accumulation in HFD-fed mice. C57BL/6J mice were fed with HFD for 12 weeks with oral administration of CGA (150 mg/kg) or metformin (200 mg/kg), and the effects of CGA on body weight, OGTT, fasting plasma glucose, PTT, food intake (n = 10), and ceramide accumulation (n = 6) in liver were analyzed (**A**–**F**). Phosphorylation of Akt, FoxO1, and p38 protein expression (**G**–**I**) was determined using western blot (n = 4). Data are expressed as the mean ± SD. * *p* < 0.05 vs. HFD, ^#^ *p* < 0.05 vs. Chow. CGA, Chlorogenic acid; Met, Metformin.

As compared to the chow-fed group, mice subjected to 12-week HFD showed increased body weight and impaired OGTT (Figure 6A,B), suggesting the successful establishment of diabetic models. Furthermore, HFD feeding enhanced hepatic glucagon response, and led to increased fasting plasma glucose (Figure 6C). HFD-fed mice showed increased PTT results compared with control mice (Figure 6D). However, the impaired OGTT, and the increased body weight, fasting plasma glucose, PTT, and ceramide level were significantly reversed in HFD-fed mice supplemented with CGA without significant influence on food intake (Figure 6A–F). These resulted in the activation of glucagon-mediated Akt dephosphorylation in the liver, enhancing the ability of glucagon to increase the translocation and activation of FoxO1 (Figure 6G,H). In contrast, CGA treatment drastically lowered ceramide accumulation induced by HFD, leading to reductions in the expression of Akt dephosphorylation and FoxO1 translocation. Given that the inhibition of inflammation has been suggested to play critical roles in restraining glucagon response by CGA, the effects of CGA on p38 MAPK activation were investigated. Glucagon induced a significantly higher expression of p38 MAPK protein, whereas CGA incubation effectively restored this alteration (Figure 6I).

## 4. Discussion

The interaction of lipid and glucose metabolism in the liver has been well established [21]. Among the lipids that influence the glucose homeostasis, sphingolipid metabolites, particularly ceramide, have been shown to impair the hepatic insulin signaling, leading to high blood glucose [11], while the relationship with glucagon-mediated responses has not been established. Ceramides have become particularly important signaling molecules in the inflammation response, while T2DM has long been recognized as a chronic inflammatory disease [13,14,22]. In addition, ceramide accumulation in the liver is derived from the dysregulation of lipolysis, while inflammation could induce lipolysis to enhance the delivery of free fatty acids to the liver, contributing to ceramide accumulation [11,14,21]. However, it is still unclear whether and how CGA regulates the de novo synthesis of ceramide, concomitant with the amelioration of inflammation, to improve the dysregulated glucagon response. In the present study, the impaired glucose homeostasis was significantly improved by the administration of CGA in HFD-induced diabetic mice. Notably, the inhibitory effects of CGA on ceramide accumulation are associated with the anti-inflammation activity, which ameliorates the hepatic glucagon response.

The important role of oxidative stress and inflammation in the development of T2DM has been well evidenced [7,13,15,23]. Low-grade inflammation has been observed in subjects with T2DM, along with the increase in systemic plasma proinflammatory and inflammatory cytokines such as TNF-α, IL-6, etc. NF-κB, a transcriptional factor, is involved in the activation of many genes linked to inflammation [24]. It has also been demonstrated that the levels of p38 phosphorylation are increased in type 2 diabetic adipocytes, which downregulate the translocation of insulin stimulated GLUT4, contributing to the insulin resistance [25]. In particular, the action of p38 has been implicated in hepatic glucose production, which is known for its role in the activation of inflammation [26]. The results have shown that p38 plays an essential role in the FP/CaMKIIγ-regulated nuclear translocation of FoxO1, which facilitates hepatic gluconeogenesis [27]. Of note, anti-inflammation may provide an important therapeutic target to improve systematic glucose metabolism in patients with diabetes [28]. Previous studies indicated that the phosphorylation of p38 cascade was inhibited by the administration of CGA in interferon gamma and phorbol myristate acetate-challenged Caco-2 cells [29]. The inhibition of the NOX/ROS/MAPK p38 signaling pathway that leads to the amelioration of oxidative stress could partly explain the protective effects of CGA on liver fibrosis [30]. Therefore, there seems to be great potential for the inhibition of p38 by CGA to improve hepatic glucose metabolism. We performed a PTT and GTT assay to assess the potential anti-diabetic efficacy of CGA. As predicted, our data showed that the elevated glucose levels were lowered by the administration of CGA in mice, suggesting its inhibition effect on hepatic glucose output. Furthermore, the levels of inflammatory factors including TNF-α, IL-6, and p38 activation were also reduced by the treatment with CGA in mice. These suggest that CGA improves glucose metabolism via the regulation of inflammation.

In addition to the anti-inflammation activity, CGA has been reported to improve the lipid metabolism. Saturated free fatty acids-induced oxidative stress and dysfunction of mitochondrial biogenesis in hepatocytes are ameliorated by preincubation with CGA [17]. CGA has also been reported to protect primary rat hepatocytes from palmitic acid-induced apoptosis via inhibition of endoplasmic reticulum stress [31]. Various classes of lipids, particularly ceramide, have been implicated in lipotoxicity, which underlies T2DM [11]. Indeed, obesity and overnutrition are associated with the accumulation of ceramide which activates inflammatory pathways, thereby leading to insulin resistance [11,12,28]. We demonstrated that CGA decreased the accumulation of ceramide in liver tissue, in line with the hypothesis that glucose homeostasis was improved by CGA via the regulation of ceramide synthesis. Ceramide has been widely reported to inhibit the phosphorylation of Akt. The activation of protein kinase C by ceramide can inhibit the translocation of Akt, leading to hepatic insulin resistance [32]. A recent study demonstrated that glucagon inactivated Akt and the subsequent upregulation of FoxO1, resulting in the hepatic glucose production [8]. To investigate the mechanism underlying the inhibitory action of CGA on hepatic glucagon response, the levels of Akt and FoxO1 were investigated. CGA reversed glucagon impaired activation of Akt by phosphorylation, which led to the inactivation of FoxO1.

Ceramides are mainly produced by the de novo synthesis pathway: serine and palmitoyl-CoA under the action of a series of enzymes such as serine palmitoyltransferase, CerS1-CerS6 and Degs1 [11,12]. A recent study showed that Des1-knockout mice improved insulin resistance and lipid accumulation in the liver induced by high-fat feeding by reducing ceramide accumulation, suggesting that ceramide accumulation is an important factor in the disorder of glucolipid metabolism [11]. Studies have reported that the inhibition of ceramide biosynthesis alleviated diet-induced insulin resistance [11,12,13,32,33]. However, ceramides are a class of lipids that are distinguished by their fatty acid carbon chain length and unsaturation [12]. Different ceramides have different biological activities, so the comprehensive inhibition of ceramide synthesis with Des as a target may impair the physiological function of normal cells. Six ceramide synthases have been reported to regulate the synthesis of ceramides [34,35]. Among the various ceramides with different acyl-chain length, CerS6-derived C16 ceramides contribute to metabolic disorders [36,37]. The inhibition of C16 ceramide synthesis has emerged as an effective strategy to improve glucose and lipid metabolism abnormalities in various diseases, such as diabetes, nonalcoholic steatohepatitis, cardiovascular disease, etc. The present results suggest that CGA restrained glucagon response by reducing the concentration of C16 ceramides, which preserved the Akt phosphorylation, thereby inhibiting the hepatic glucose production. Our work had a few limitations. For instance, whether HFD, glucagon, or both is responsible for promoting ceramide accumulation and hepatic gluconeogenesis was not directly proved, which could be further confirmed by determining the effects of glucagon on PEPCK, G6pase, TNFa, IL-6, and p38 in HFD-fed mice. A long-term ceramides feeding is required to verify the role of ceramides on hepatic glucose production.

## 5. Conclusions

We investigated the protective effects of CGA on glucose and ceramide metabolism both in vivo and in vitro. In conclusion, the present results suggested that CGA inhibited glucagon-induced endogenous glucose production, which is associated with the suppression of the accumulation of ceramides. CGA attenuated the inflammation response and ceramide accumulation in the liver of HFD-induced diabetic mice. Treatment with CGA protected Akt phosphorylation, subsequently inhibiting the nuclear translocation of FoxO1. We provided new insights into the cellular basis for CGA in the regulation of glucagon-induced hepatic gluconeogenesis in diabetes.

## Figures and Tables

**Table 1:** Primer pairs for qRT-PCR.

Gene		Sequence 5′–3′
*CerS5* (mice)	Forward	TCAGACTTCTTGCTGGAGGT
	Reverse	AGAGTCCACAGAAGGTCAGC
*CerS6* (mice)	Forward	AGAACTTGAGGATTGGAGGGTT
	Reverse	CTGACAACACGTTCTCCAGC
*Degs1* (mice)	Forward	GAATGGGTCTACACGGACCAG
	Reverse	CGAGAAGCATCATGGCTACAA
*G6pase* (mice)	Forward	CGACTCGCTATCTCCAAGTGA
	Reverse	GTTGAACCAGTCTCCGACCA
*PEPCK* (mice)	Forward	AAGCATTCAACGCCAGGTTC
	Reverse	GGGCGAGTCTGTCAGTTCAAT
*β-actin* (mice)	Forward	AGTGTGACGTTGACATCCGTA
	Reverse	GCCAGAGCAGTAATCTCCTTCT

## Data Availability

The datasets used and/or analyzed during the current study are available from the corresponding author upon reasonable request.

## Supplementary Materials

The following supporting information can be downloaded at: https://www.mdpi.com/article/10.3390/nu15143173/s1, Table S1: Antibodies for immunoblotting.

## Abbreviations

AUC	Area under the curve
CerS6	ceramide synthetase 6
CGA	Chlorogenic acid
Degs1	Dihydroceramide desulfurase 1
G6pase	Glucose-6-phosphatase
GTT	Glucagon tolerance test
HFD	High-fat diet
OGTT	Oral glucose tolerance test
PEPCK	Phosphoenol pyruvate carboxykinase
PTT	Pyruvate tolerance test
T2DM	Type 2 diabetes mellitus
