# Supplementary material for: Chlorogenic Acid Inhibits Ceramide Accumulation to Restrain Hepatic Glucagon Response
Source: Nutrients. 2023 Jul 17;15(14):3173. doi: 10.3390/nu15143173 (PMC10384019; doi:10.3390/nu15143173)
Supplement: Supplementary file 1 [file nutrients-15-03173-s001.zip › nutrients-2482072-supplementary.pdf]

Supplementary Table S1. Antibodies for immunoblotting.

Antibody	Cat No.	Company	Dilutions	Source
FOXO1	2880s	CST	1:1000	Rabbit
p- FOXO1	9461s	CST	1:1000	Rabbit
Akt	CY5561	Abways	1:1000	Rabbit
p-Akt	CY6569	Abways	1:1000	Rabbit
p-P38	CY6391	Abways	1:1000	Rabbit
P38	CY5262	Abways	1:1000	Rabbit
β-actin	AB0035	Abways	1:4000	Rabbit
Sencondary Antibody	#L3012	SAB	1:8000	Rabbit
